# Synthesis, Controlled Release, and Stability on Storage of Chitosan-Thyme Essential Oil Nanocapsules for Food Applications

**DOI:** 10.3390/gels7040212

**Published:** 2021-11-14

**Authors:** Ricardo M. González-Reza, Humberto Hernández-Sánchez, David Quintanar-Guerrero, Liliana Alamilla-Beltrán, Yair Cruz-Narváez, María L. Zambrano-Zaragoza

**Affiliations:** 1 Departamento de Ingeniería Bioquímica, Escuela Nacional de Ciencias Biológicas, Instituto Politécnico Nacional, Unidad Profesional Adolfo López Mateos, Ciudad de México C.P. 07738, Mexico; gonzalez.reza@comunidad.unam.mx (R.M.G.-R.); hhernan1955@yahoo.com (H.H.-S.); lalamilla@ipn.mx (L.A.-B.); 2 Laboratorio de Procesos de Transformación y Tecnologías Emergentes de Alimentos, FES-Cuautitlán, Universidad Nacional Autónoma de México, Cuautitlán Izcalli C.P. 54714, Mexico; 3 Laboratorio de Investigación y Posgrado en Tecnología Farmacéutica, FES-Cuautitlán, Universidad Nacional Autónoma de México, Cuautitlán Izcalli C.P. 54740, Mexico; quintana@unam.mx; 4 Laboratorio de Posgrado de Operaciones Unitarias, Escuela Superior de Ingeniería Química e Industrias Extractivas, Instituto Politécnico Nacional, Unidad Profesional Adolfo López Mateos, Ciudad de México C.P. 07738, Mexico; ycruzn@ipn.mx

**Keywords:** antioxidant capacity, diffuse reflectance, food nanotechnology, emerging technologies

## Abstract

The nanoencapsulation of thyme essential oil has been greatly important in food science, given its remarkable antioxidant and antimicrobial capacity. However, its analysis in storage has not been established in terms of physical stability, antioxidant capacity, and release studies. In this paper, chitosan-thyme oil nanocapsules were prepared by the ionic gelation method. These were characterized for differential calorimetry, release kinetic, and infrared spectroscopy. The chitosan-thyme oil nanocapsules were stored at 4 and 25 °C for 5 weeks, the changes in particle size, zeta potential, stability (diffuse reflectance), and antioxidant capacity were analyzed and associated with nanocapsules’ functionality. The results show that the storage time and temperature significantly modify the particle size (keeping the nano-size throughout the storage), the release of the bioactive was Fickian with t^0.193^ according to Korsmery & Peppas and best described by Higuchi model associated with changes in the zeta potential from 8 mV to −11 mV at 4 °C. The differential scanning calorimetry and infrared spectroscopy results confirm the good integration of the components. The antioxidant capacity revealed a direct relationship with residual oil concentration with a decrease in the ABTS test of 15% at 4 °C and 37% at 25 °C. The residual bioactive content was 77% at 4 °C and 62% at 25 °C, confirming nanoencapsulation effectiveness. The present investigation provides helpful information so that these systems can be applied in food conservation.

## 1. Introduction

Nanotechnology is one new technology that has emerged to impact food science. This approach focuses on designing, characterizing, producing, and applying systems and components of submicron size (1 nm = 1 × 10^−9^ m) to form substances or materials with specific, desirable properties [1]. Polymeric nanoparticles are defined as colloidal particles whose main characteristic is their size: from 1 to 500 nm [2]. Nanocapsules (NC) are vesicular systems made of polymeric membrane or wall, in which the active molecules are encapsulated [3]. The nanoencapsulation of lipophilic substances has made it possible to provide improved properties to various essential oils, increasing the solubility and protection against light, pH, oxygen, among others [4]. The wall polymer should be preferer of natural origin with the advantage of having the best compatibility with food components, and to have the functionality desired in the system, consider the polymer compatibility with the compound to be transported [5].

Chitosan (CS) is a natural polymer obtained from the deacetylation of chitin from the exoskeleton of crustaceans; its use has a significant impact on the environment and has been shown to have significant antimicrobial and antioxidant capacity [6,7]. Among the attractive bioactive substances for use in food processing are essential oils, which have significantly impacted food processing, given their antioxidant and antimicrobial properties [8]. One of the essential oils has captured the attention in food processing is thyme essential oil (TEO); it has shown antimicrobial activity and potent antioxidant capacity due to molecules such as thymol, carvacrol, β-caryophyllene, γ-terpinene ρ-cymene, among others [9]. However, being an essential oil tends to volatilize easily, and given its lipophilic origin, it has a disadvantage when incorporated in aqueous food matrices.

Nanoencapsulation of TEO has been investigated; however, the stability as time and storage temperature is not yet in sight. Several studies focus on the nanoencapsulation of various essential oils; most of the efforts are focused on finding the optimal preparation conditions as an effect of encapsulating, stabilizing polymers and aspects of antioxidant parameters and antimicrobials [10,11,12]. In this sense, numerous investigations report the nanoencapsulation of thyme essential oil in chitosan as a polymeric matrix [13,14,15]. However, the kinetic, antioxidant, and physical stability changes given by storage time and temperature conditions have not been thoroughly evaluated.

The objective of this research was to obtain nano-encapsulated thyme essential oil and study their functionality during the storage at 4 and 25 °C for 5 weeks in order to establish the application in different food technology areas as edible coatings, active packaging, and development of functional foods. In addition, the functionality was evaluated about the antioxidant capacity, stability, changes in particle size, polydispersity, and zeta potential. Furthermore, the controlled release kinetic was obtained to explain the mechanisms or release of thyme oil from the core of the nanocapsule. Evaluating these parameters is considered of vital importance for applications in food processing and functional food development.

## 2. Results and Discussion

### 2.1. Dynamic Light Scattering (DLS) and Electrophoretic Movement (ζ)

Table 1 shows the particle size (PS)from initial characterization on chitosan nanoparticles (CSNP) and chitosan-thyme essential oil nanocapsules (TEO-CSNC); these had unimodal comportment and sizes <150 nm. The increase in the PS of the TEO-CSNC in contrast to the NE was not significant (*p* > 0.05). The polydispersity index (PDI) of both systems was ≤0.3, indicating a narrow size distribution [16]. The zeta potential (ζ) of the nanoemulsion is negative due to the functional groups in TEO; it becomes positive due to amino groups from chitosan.

### 2.2. Encapsulation Efficiency (EE) and Release Kinetics

**Figure 1:**
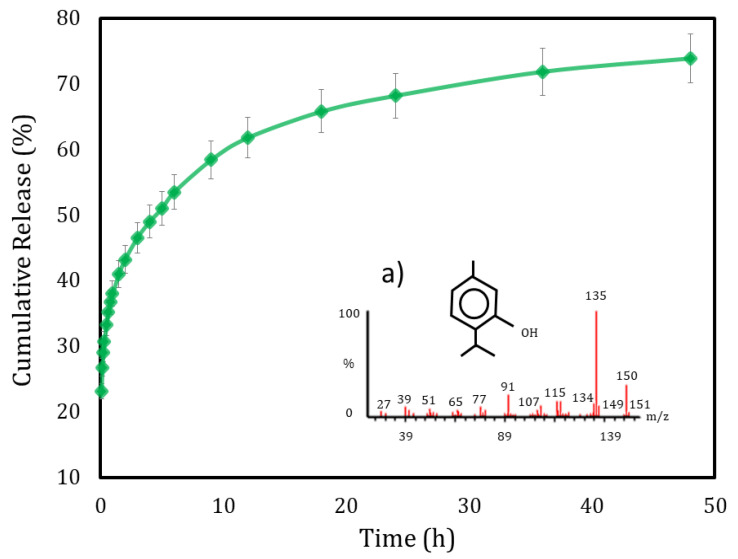
In vitro release profile obtained for the chitosan-thyme nanocapsules, (a) mass spectrum of thymol found in thyme essential oil (majority compound in the sample).

The EE of TEO-CSNC was 69.38 ± 3.15%; this content agrees with the report to thymol and carvacrol components with EE of 46.3% and 50.9% in nano-cochleates and 68% on NC of chitosan with TEO [17,18]. Figure 1 shows the TEO-CSNC release profile, observing that near to 60% of TEO was released at 9 h.

Table 2 shows the constants and coefficients of models of release, and the semiempirical model of Korsmeyer-Peppas with a value of “n” exponential <0.5, suggesting a Fickian release mechanism dependent on the time t^0.193^, which can be explained by TEO diffusion across the membrane, possible swelling, and breaking film [19].

Surprisingly, good R^2^ values were obtained for the Higuchi model, which applies to matrixial systems; apparently, a pseudo-stable constant gradient formation does not apply to this capsular system. Thyroid essential oil release studies have reported similar results for chitosan nanocapsules prepared by nanoprecipitation [20]. The equation to describe the release of TEO is:

Oil released (%) = 37.73 × Time (h)^0.193^

### 2.3. Scanning Electron Microscopy (SEM)

**Figure 2:**
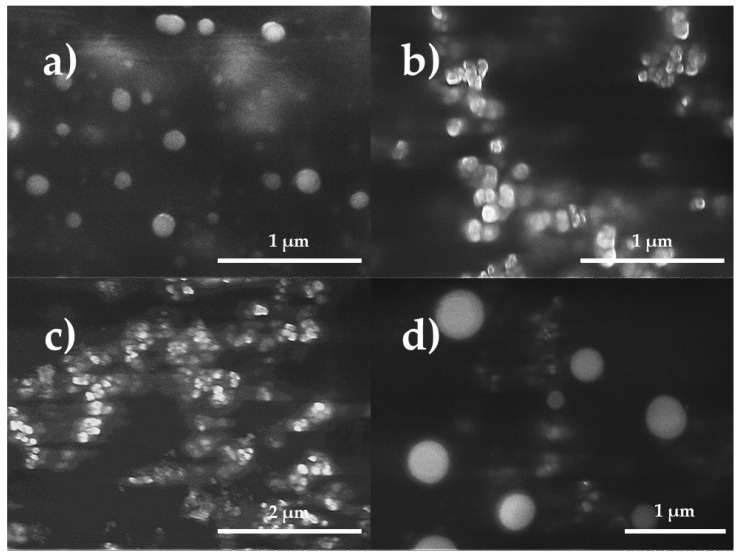
Morphological characterization by SEM of the nanocapsules (**a**) without charge, (**b**) loaded with thyme essential oil, (**c**) loaded with active in week 5 of storage at 25 °C, and (**d**) loaded with thyme essential oil in week 5 of storage at 4 °C.

Figure 2 shows the micrographs of the CSNP and TEO-CSNC; these were of a nanometric size and spherical and regular shape with any agglomerations. The sphere structure consists of the polymeric matrix that traps the essential oil. The average size of the TEO-CSNC observed by SEM was successfully correlated with the average size obtained by laser light scattering.

### 2.4. Differential Scanning Calorimetry (DSC)

**Figure 3:**
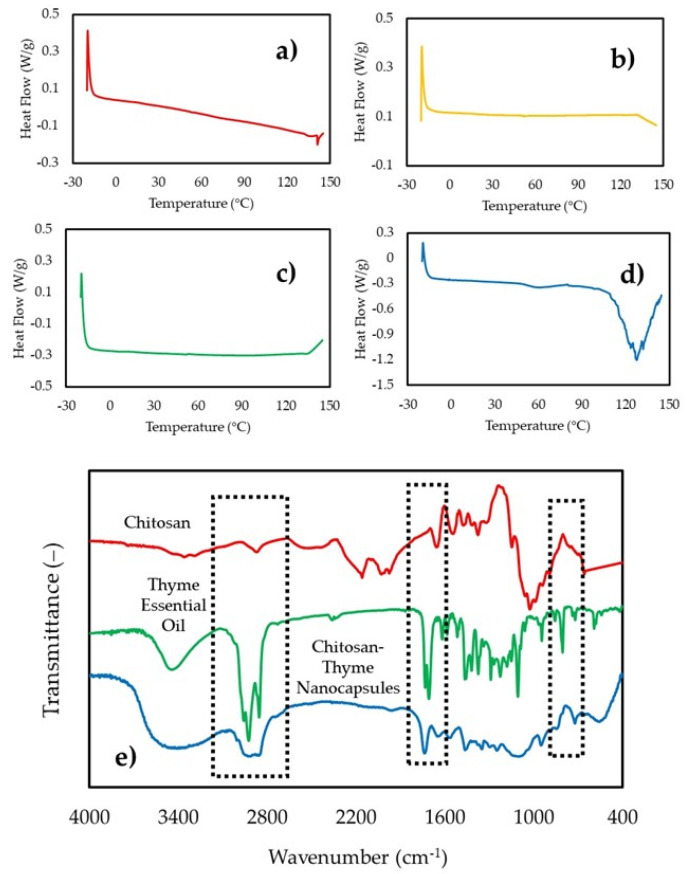
Differential scanning calorimetry for the components of the nanocapsules (**a**) chitosan, (**b**) TPP, (**c**) thyme essential oil, and (**d**) chitosan-thyme nanocapsules, and (**e**) infrared spectra of the chitosan nanocapsules obtained by ionic gelation and its components.

Figure 3 shows the DSC thermograms. Figure 3a shows the heat flow obtained for chitosan, with a melting peak at 141 °C, consistent with studies reported by [6]. No significant changes in heat flow were observed between 20 and 150 °C, and no hydrolysis or oxidation of TEO components was seen (*p* > 0.05) by tripolyphosphate used as a crosslink agent. Figure 3d shows the CSNP cross-linking with TPP melting at 127.52 °C lower than the pure chitosan; TEO-CSNC has a ΔH of 82.17 kJ/kg Tg of 102.35 °C, indicating physical integration of the components.

### 2.5. Infrared Spectroscopy (IR)

The IR spectroscopy was applied to confirm the cross-linking between the nanostructures of chitosan and the TEO. The IR spectrum of all the components is presented in Figure 3e, showing their typical bands: at 3357 cm^−1^_,_ the O-H stretch superimposed on the H stretch bands; in 2874 cm^−1^_,_ the stretching of the C-H; the amide I band can be found at 1649 cm^−1^ (C-O stretch of the acetyl group) and the amide II band at 1587 cm^−1^ (N-H flex). The sharp bands at 1415 cm^−1^, 1374 cm^−1^, and 1316 cm^−1^ attributed to the flexion of CH_2_, the asymmetric flexion of the CH of the CH_2_ group, and the amide band III, respectively [13].

Typical absorption peaks of TEO were detected in the nanocapsules at 2926 cm^−1^ and 2969 cm^−1^ in all samples. The peaks at 1563 cm^−1^ and 1466 cm^−1^ correspond to the vibration C=C of the benzene ring in the oil loaded in the nanocapsule. The peaks at 1374 cm^−1^ and 1110 cm^−1^ are attributable to the flexion mode C-O-H and the stretching vibration -C-O-, respectively. The peak at 947 cm^−1^ relates to C=C stretching vibration or the flexural vibration of some hydrogen-containing groups, confirming the interaction between chitosan nanostructured and TEO.

### 2.6. Dynamic Light Scattering (DLS) during the Storage

The PS evolution is shown in Table 3; all the samples had PS ≤ 300 nm. The ANOVA revealed a significant effect due to temperature and storage time (*p* ≤ 0.05). The PS increase is explained as a swelling of the polymer matrix, and the TEO was released and left exposed very close to the CSNC surface, which correlates directly with the release results discussed above. Studies conducted for the encapsulation of essential oils indicate similar values in the PS ≥ 500 nm such as cinnamon and clove oil [21,22]. The decrease in PS after 3 weeks results from a greater packing of the polymer chains due to the high number of amino groups in the chitosan responsible for the interaction with TEO [21].

Table 3 shows the evolution in the TEO-CSNC on PDI during storage, observing the significant effect of the storage (*p* ≤ 0.05) the aggregation phenomena can increase. However, the principals’ PDI changes were present at 3 and 4 weeks in samples at 4 and 25 °C, respectively, When the TEO was released. However, there is no research on CSNC behavior during storage and the greater stability at 25 °C attributing to the relaxation of the polymer chains, which make the components of the system not had large aggregation of particles.

### 2.7. Electrophoretic Movement (ζ) in the Storage

Table 3 shows the ζ evolution of CSNC-TEO. The zeta potential obtained for the chitosan nanocapsules without TEO was 31.8 ± 2.8 mV. The ζ reduction was associated with the fact that the positively charged amino groups bind to the OH groups of the thymol and the carvacrol contained in the thyme essential oil. The increase observed in the average size, together with the decrease in zeta potential, have also been reported after loading carvacrol, eugenol, and lime essential oils, and ellagic acid into chitosan nanoparticles [21]. Similar values have been reported for eugenol-chitosan nanoemulsions produced by ultrasound-mediated emulsification [23]. After 3 weeks at 4 °C the ζ decreases and shows a statistically significant difference (*p* ≤ 0.05). This compartment is explained by the low availability of NH_2_ groups from chitosan; since these interact with the thyme oil release across the OH groups present in the phenols and aromatic compounds [20]. The functional groups that interact are visible in the infrared presented in Figure 3.

### 2.8. Thyme Essential Oil Residual Content

The TEO concentration is shown in Table 3; the ANOVA evidences a statistically significant effect of storage time and temperature (*p* ≤ 0.05). A significant decrease occurred in the samples stored at 25 °C in the first week of storage, as they presented losses of the active agent of approximately 20%. This can be explained by the fact that the terpenes of the essential oils are volatile compounds, so the effect of temperature was a determining factor in their residual concentration [8]. Stabilization of the thyme essential oil concentration occurred in storage at 4 °C (~75%) from week 2 until the end of storage, in contrast to the result of ~60% obtained at 25 °C. Numerous studies mention the effect of temperature on thermolabile compounds during storage. However, the polymer structure affects the properties of bioactive agents including the antioxidant capacity discussed below.

### 2.9. Antioxidant Capacity

#### 2.9.1. ABTS

**Figure 4:**
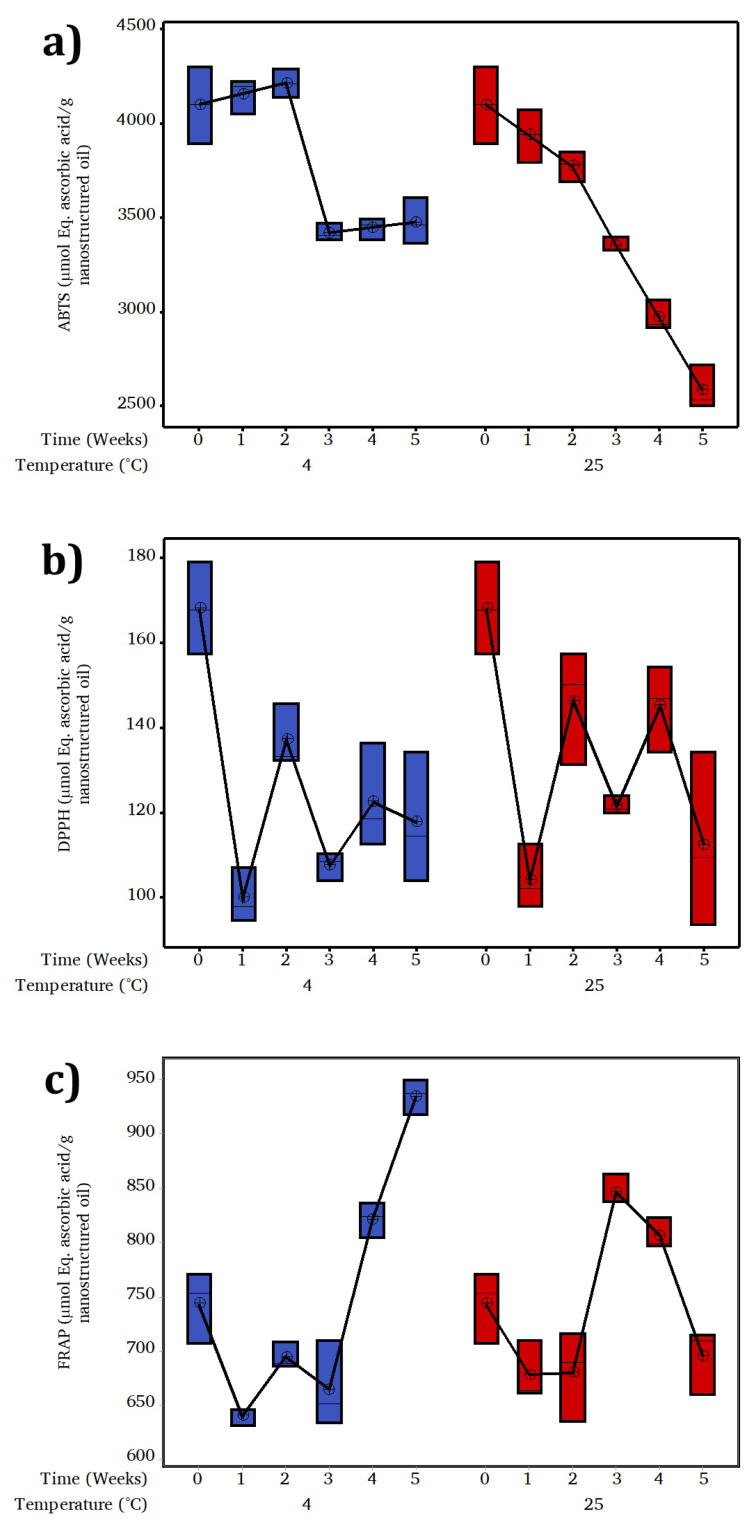
Evolution of antioxidant capacity by: (**a**) ABTS; (**b**) DPPH; and (**c**) FRAP of chitosan-thyme nanocapsules onto storage at 4 and 25 °C.

The antioxidant activity determined by ABTS of the TEO was 2238 µmol Eq ascorbic acid/g of TEO that contrasts to that obtained in the chitosan TEO-CSNC, which was 4104 µmol Eq ascorbic acid/g of TEO-CSNC. This difference is because chitosan has an excellent antioxidant capacity [7,24], denoted by the significant effect of chitosan that promotes ABTS oxidation due to its ability to donate electrons. Figure 4a shows the ABTS, which is directly proportional to the total active agent concentration in the samples analyzed as a function of time.

The antioxidant capacity decreases 16% in the third week at 4 °C no further degradation until the end of storage (~3300 µmol Eq ascorbic acid/g of TEO-CSNC); so, under this temperature, it is possible to maintain the antioxidant capacity and to have functionality for application in food preservation. The TEO-CSNC at 25 °C decreases the ABTS by ~50% at the end of storage (~2582 µmol Eq ascorbic acid/g of TEO-CSNC) (*p* ≤ 0.05). Attributable to the temperature, at 25 °C the polymer chains modify the permeability and facilitate TEO release, that oxidized quickly under the environmental condition; however, its application in food preservation is viable until the third week else it loses 18% of its antioxidant capacity. A similar phenomenon was reported by [25], showing that the activity of sweeping ABTS radicals from chitosan films with incorporated ferulic acid has a time-dependent behavior.

#### 2.9.2. DPPH

Figure 4b shows the antioxidant activity by DPPH. The antioxidant activity of TEO was different (*p* ≤ 0.05) from that obtained for TEO-CSNC with a value of 274.74 µmol Eq ascorbic acid/g of oil 176.9 µmol Eq ascorbic acid/g of TEO-CSNC, respectively. A significant decrease (*p* ≤ 0.05) occurred in the first week of temperature-independent storage. Subsequently, no statistically significant differences were found given by temperature or storage time (*p* > 0.05) (~130 µmol Eq ascorbic acid/g of TEO-CSNC); this may be the result of the DPPH radical being removed by the Maillard reaction with its reaction products by developing reductants that terminate the free radicals. Therefore, the Maillard reaction between the hydroxyl group and the OH groups of the TEO molecules is more effective in capturing DPPH radicals than the amino groups of chitosan, as described in detailed experiments [26] for chitosan-ascorbate nanoparticles. The results of the ABTS trial showed congruence with the DPPH method. The increase in TEO concentration has progressively eliminated the ABTS radical determined by ABTS discoloration and the DPPH radical.

#### 2.9.3. FRAP

Unlike DPPH and ABTS, FRAP measures the reducing (chelating) power of the samples. The results obtained are shown in Figure 4c. The antioxidant capacity by FRAP of TEO was different (*p* ≤ 0.05) from the TEO-CSNC with 946.04 µmol Eq ascorbic acid/g of TEO and 746.8 µmol Eq ascorbic acid/g TEO-CSNC, respectively. There was no statistically significant difference in the FRAP-values until the third week at 4 °C and the second week at 25 °C after having an increase in antioxidant capacity by FRAP (*p* ≤ 0.05), however at 25 °C, in the end, it showed a decrease due to the oxidation of TEO which correlates directly with the release profiles previously analyzed, in addition to the ζ values determined for the last weeks of storage at 4 °C. Studies conducted by [27] for films composed of chitosan and phenolic extracts showed that they attribute the values of antioxidant capacity to the release of bioactive compounds, which coincides with that obtained in this study.

### 2.10. Diffuse Reflectance

**Figure 5:**
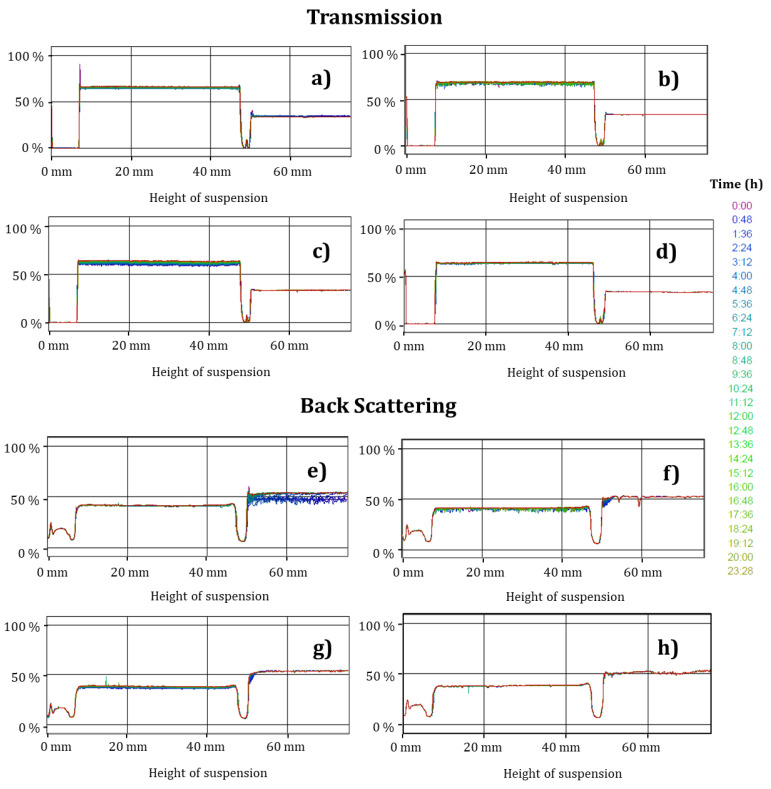
Transmission and backscatter profiles of nanocapsules stored at 25 °C. (**a**,**e**) initial, (**b**,**f**) week 1, (**c**,**g**) week 3 (**d**,**h**) week 5.

**Figure 6:**
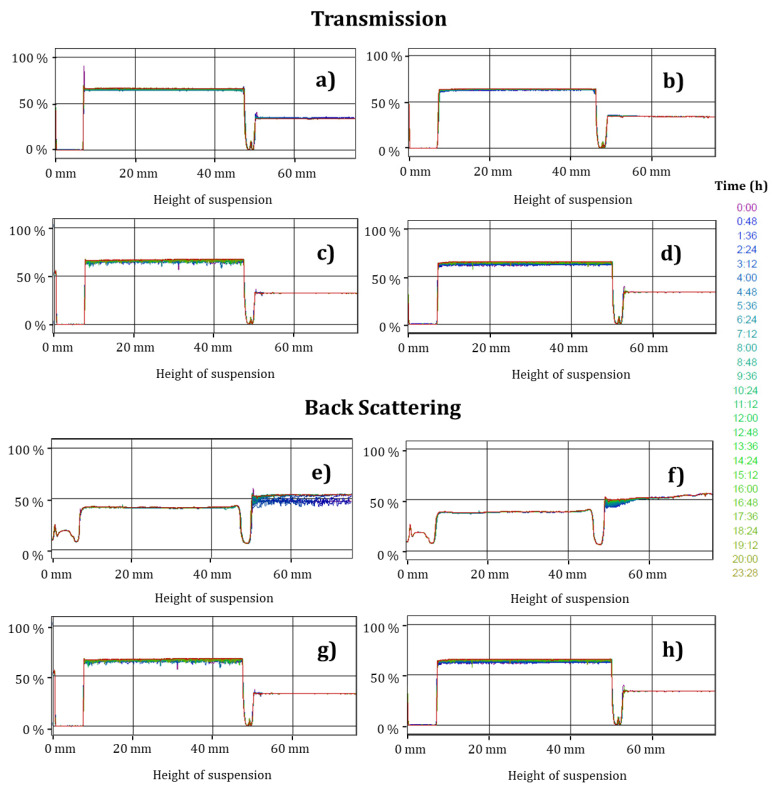
Transmission and backscatter profiles of nanocapsules stored at 4 °C. (**a**,**e**) initial, (**b**,**f**) week 1, (**c**,**g**) week 3, (**d**,**h**) week 5.

Figure 5 and Figure 6 show the changes in the transmission and backscattering profiles of the TEO-CSNC at 25 and 4 °C, respectively.

The samples do not have statistically significant differences (*p* > 0.05) in terms of transmission, backscatter profiles, and maximum thickness given by storage time and temperature. A slight destabilization was found at the end of storage at 4 °C (Figure 6d,h), correlating with TEO release and changes in the ζ. However, this was not significant (*p* > 0.05). It was attributed to the fact that the released oil still retained its nanometric size and was stabilized through charges in the aqueous medium.

## 3. Materials and Methods

### 3.1. Materials

Thyme essential oil (ρ = 0.917 g/cm^3^ at 25 °C) from Sigma-Aldrich^®^ (St. Louis, MO, USA) (Lot: MKCF4333) is used to form the oil nucleus. Chitosan (MW = 50–190 kDa, μ = 20–300 cP in CH_3_COOH solution (1 g/L) at 25 °C) (Lot: STBH6262) as the encapsulating polymer, Tween^®^ 80 and Span^®^ 80 (Lot: MKBV4425V) were purchased from Sigma Aldrich^®^. The distilled water was Milli-Q^®^ grade (Millipore Corporation, Bedford, MA, USA). ABTS (2,2′-azino-bis- (3-ethylbenzthiazoline)-6-sulfonic acid) (Lot: SLBD8908V), DPPH (2,2-diphenyl-1-picrylhydracil) (Lot: STBG9431), FRAP (2,4,6-tri (2-pyridyl)-s-triazine) (Lot: BCBW6989) were purchased from Sigma Aldrich^®^ (St. Louis, MO, USA). Ascorbic acid (C_6_H_8_O_6_) (Lot: A0616289), potassium persulfate (K_2_S_2_O_8_) (Lot: M1010511), potassium dihydrogen phosphate (KH_2_PO_4_) (Lot: M0918575), dipotassium hydrogen phosphate (KH_2_PO_4_) (Lot: M1014575), and glacial acetic acid (CH_3-_COOH) (Lot: A0414235) were purchased from Meyer^®^ (Reactivos Química Meyer, Ciudad de México, México). Cyclohexane (C_6_H_12_) and methanol (CH_3_OH) (Lot: 824333) were purchased from Fermont^®^ (Productos Químicos Monterrey, México).

### 3.2. Nanoemulsion Preparation (NE)

The thyme essential oil-nanoemulsion (TEO-NE) was prepared by the ultra-high agitation method [28]. Briefly, 5 g/L of Span^®^ 80 and 2 g/L of TEO were added to the dispersed phase. The continuous phase was composed of 10 g/L Tween^®^ 80. The NE was obtained in 3 cycles of 5 min at 1047.2 s^−1^ of ultra-high agitation and resting intervals of 5 min between cycles, using a rotor/stator homogenizer (Ultra-Turrax T25, IKA^®^, Wilmington, DC, USA) at 25 °C. This NE was the basis for the formation of chitosan nanocapsules.

### 3.3. Chitosan Nanocapsules Preparation (CSNC)

The CSNC were prepared by the ionic gelation method. Briefly, 50 mL of the NE prepared above was added to glacial acetic acid (1 g/L) and chitosan (3 g/L) to prepare a dispersion by magnetic stirring at 78.54 s^−1^ for 2 h. Once the dispersion was completely homogeneous, it was maintained with magnetic stirring at 78.54 s^−1^ at 25 °C, and 20 mL of sodium tripolyphosphate (3 g/L) solution was added dropwise (1 mL/min) using a syringe pump (NE-1000, New Era Pump Systems Inc. Farmingdale, New York, NY, USA). Once the components were mixed, ultrasonication was applied for 3 min utilizing an ultrasonic processor (Hielscher Ultrasonics, UP200Ht, Teltow, Germany) with a titanium sonotrode (Hielscher Ultrasonics, S26d14, Teltow, Germany) with a diameter of 14 mm (154 mm^2^), 80 mm in length and an external thread, at a power of 50 W later to adjust the pH of the mixture at 5 with NaOH (0.2 N). Once obtained, the samples are stored in 100 mL high-density polyethylene terephthalate containers at 4 and 25 °C for analysis every 7 days for 5 weeks. Chitosan nanoparticles (CSNP) were prepared in the same form without TEO- NE and used as a comparison test.

### 3.4. Dynamic Light Scattering (DLS) and Electrophoretic Movement (ζ)

The particle size (PS) and the polydispersity index (PDI) were determined by laser-scattering using a Z-sizer 4 (Zetasizer Nano Series, Malvern Ltd., Enigma Business Park, Grovewood Road, UK) at a 90° angle, for the nanosystems. The zeta potential (ζ) was determined by electrophoretic mobility normalized with standard polystyrene dispersion (ζ = –55 mV) using a Z-sizer Nano ZS90 (Malvern Ltd., Enigma Business Park, Grovewood Road, UK). For both the determinations, the samples were diluted with Milli^®^ Q distilled water in a ratio of 1:20. The measurements were made at 25 °C in triplicate [29].

### 3.5. Encapsulation Efficiency (EE) and Release Kinetics

The encapsulation efficiency (EE) was determined by spectrophotometry using a Genesys 10 s UV/VIS spectrophotometer (Thermo Scientific, Waltham, Massachusetts, USA) at 275 nm separating the quantity not encapsulated by centrifugation (Hermle Z323K, Labortechnik GMBH, Wehingen, Germany) for 20 min at 4 °C at 18,000× *g*. The release studies are carried out according to the methodology proposed by [19]. Briefly, TEO-CSNC was lyophilized, and the powder resuspended in 1 g/L of cyclohexane after centrifugation at 18,000× *g* for 20 min at 4 °C, and the release profiles were obtained to quantify the thyme oil expelled from nanocapsules in the supernatant. The volume lost periodically for each measurement was replaced. The absorbance was measured in a Genesys 10 s UV/VIS spectrophotometer (Thermo Scientific, Waltham, MA, USA), and the concentrations of active release were obtained with respect to the TEO calibration curve at 275 nm. The controls were made by centrifuging 1 mL of free polymer in the release medium. All release experiments of the nanocapsules were performed in triplicate for 48 h at 25 °C [30].

### 3.6. Scanning Electron Microscopy (SEM)

The TEO-CSNC and CSNP were purified by three centrifugations at 18,000× *g* for 20 min at 4 °C (Hermle Z323K, Labortechnik GmbH, Wehingen, Germany). Then a drop of the concentrated suspension was spread on a glass surface and allowed to dry. Electron channeling contrast images were taken using a high-resolution cold field emission scanning electron microscope (Hitachi, SU-8230, Tokyo, Japan), with a BSE + BSE (U) detector, an acceleration voltage of 2.5 kV, average deceleration mode with a voltage of 1.5 kV. The emission current was 5 µA, and the working distance = 3.7 mm.

### 3.7. Differential Scanning Calorimetry (DSC)

The TEO-CSNC and individual components were evaluated using a differential scanning calorimeter (TA Instruments, DSC Discovery, New Castle, Delaware, USA) to obtain thermal comportment according methodology proposed by [30]. All samples (3 mg) were placed in hermetically sealed aluminum containers and placed in the chamber of the equipment at room temperature. Total of 40 mL/L flow of N_2_ gas was used in the purge line to control the local environment around the sample. The temperature was established in a range of –20 to 150 °C with a heating ramp of 10 °C/min. The data were analyzed with the universal analysis software provided together with the DSC instrument.

### 3.8. Infrared Spectroscopy (IR)

The individual compounds and the lyophilized CSNC were examined by spectroscopy on an IR spectrum (PerkinElmer Spectrum 400 IR, Waltham, MA, USA). Samples with minimum moisture content were placed in the prism for further analysis. The spectra were obtained with a range of 500 to 4000 cm^−1^ with a resolution of 1 cm^−1^ at 25 °C. The reference was a spectrum of ambient according to the methodology proposed by [31].

### 3.9. Diffuse Reflectance

The stability of the TEO-CSNC was determined with a Turbiscan MA2000 (Formulaction, Toulouse, France). The suspensions (5 mL) were transferred to a cylindrical flat glass sample cell for measurement. The samples destabilization was analyzed using transmission and backscattering profiles by applying pulses from an infrared light source at 880 nm every 8 min for 24 h, performing the measurement every 7 days, for 5 weeks, having two synchronous detectors: a transmission detector and a detector backscatter. The transmission detector will receive the light that moves through the sample (at 0° of the incident beam), while the backscatter detector will receive the light that is dispersed backwards through the sample (at 135° of the incident beam) [32]. The samples were analyzed at 25 °C.

### 3.10. Antioxidant Capacity

#### 3.10.1. ABTS

The antioxidant capacity by ABTS (2,2′-azino-bis- (3-ethylbenzthiazoline)-6-sulfonic acid) was determined by the methodology proposed by [30,33]. The ABTS radical cation stock solution was prepared to mix potassium persulfate solution and ABTS solution at 7 mmol and 2.45 mmol. The mixture was kept in dark at 25 °C for 16 h before its use. The working ABTS solution was obtained by dilution in phosphate buffer of pH 7.4 of the stock solution to achieve an absorbance value of 0.7 (±0.02) at 734 nm. A sample aliquot (100 µL) was added to the ABTS (2.9 mL) working solution. Absorbance was measured using a BioSpectrometer^®^ (Eppendorf AG, 22331, Hamburg, Germany) at 734 nm after 6 min. For the antioxidant capacity assay (ABTS) a standard reference curve was constructed by plotting absorbance values against concentrations of 100–1000 μmol of ascorbic acid. The analysis was done in triplicate at 25 °C.

#### 3.10.2. DPPH

The kidnapping capacity of DPPH (2,2-diphenyl-1-picrylhydracil) was determined by the methodology proposed by [30,34]. Briefly, a solution containing 100 μmol of DPPH (in methanol) was prepared. An aliquot of sample (100 µL) was mixed with DPPH solution (2.9 mL) in methanol. The mixture was vigorously stirred and incubated at 25 °C for 30 min in the dark. The absorbance was measured at 517 nm in a BioSpectrometer^®^ (Eppendorf AG, 22331, Hamburg, Germany). The antioxidant capacity assay (DPPH) a standard reference curve was constructed by plotting absorbance values against concentrations of 100–1000 μmol of ascorbic acid. The analysis was done in triplicate at 25 °C.

#### 3.10.3. FRAP

The antioxidant capacity of TEO-CSNC was evaluated by its iron reduction capacity (FRAP assay), according to the method described by [30,35]. An aliquot of TEO-CSNC (100 µL) was incubated in triplicate with FRAP reagent (containing 2,4,6-tri (2-pyridyl)-s-triazine and FeCl_3_) and distilled water at 37 °C (2.9 mL). The absorbance values were read at 595 nm on a BioSpectrometer^®^ (Eppendorf AG, 22331, Hamburg, Germany) after 30 min. A control without a sample was prepared. For the antioxidant capacity assays (FRAP) a standard reference curve was constructed by plotting absorbance values against concentrations of 100–1000 μmol of ascorbic acid. The analysis was done in triplicate at 25 °C.

### 3.11. Essential Oil Content

A Perkin Elmer gas chromatography (GC) Clarus 600 (Perkin Elmer, Inc., Waltham, MA, USA) with an Elite-5 MS column (30 m × 0.32 mm × 0.25 μm) equipped with a PerkinElmer Clarus 600 T mass spectrometer (MS) (Perkin Elmer, Inc., Waltham, Massachusetts, USA) with TurboMass 5.4.2 software was used. About 3 μL of the solution was injected, and the system was run with helium as carrier gas with a column flow of 1 mL/min. The oven was programmed to start at 50 °C. It was held at 50 °C for 5 min and then heated at 8 °C/min to a final temperature of 180 °C and kept for 5 min. Mass spectra were recorded under electron ionization (70 eV) with the m/z range: 40–550 au. Peak identification was carried out with the mass spectral library.

### 3.12. Statically Analysis

To carry out the comparison of results concerning the treatments and as a function of time, an analysis of variance (ANOVA) was performed to evaluate the significant differences (α = 0.05) between the independent variables (temperature and storage time). All the experiments were carried out considering three replicates and in random order. All statistical analyzes were performed using the Minitab statistical program (Minitab^®^ Statistical Software 19 Inc., Centre, PA, USA).

## 4. Conclusions

The infrared spectroscopy and differential scanning calorimetry characterization revealed the excellent integration of components in the nanocapsules of thyme essential oil, and scanning electron microscopy evidenced nanocapsule spherical structure. The nanocapsules of thyme essential oil prepared by ionic gelation showed good stability to storage after 5 weeks of storage at 4 and 25 °C, the lower residual content of thyme essential oil was at 25 °C. The release mechanism was Fickian according to Korsmery-Peppas with an “n” value of 0.193, and the Higuchi model was the one that best described the release of thyme essential oil from chitosan nanocapsules, thus, accepting the hypothesis raised in the present experimental work. The antioxidant capacity of nanocapsule of thyme essential oil confirms the active agent’s-controlled release in the aqueous medium, supported by the obtained values of zeta potential, the residual content of essential oil, and the release kinetics characteristic. Besides, the nanostructured systems obtained can be used without problems in the formulation and processing of foods.

## Figures and Tables

**Table 1:** Characterization by dispersion of laser light and electrophoretic movement of colloidal nanosystems.

Sample	PS (nm)	PDI (-)	ζ (mV)
Thyme Essential Oil-Nanoemulsion (TEO-NE)	123 ± 3	0.34 ± 0.01	–33 ± 3.8
Chistosan Nanoparticles (CSNP)	115 ± 3	0.265 ± 0.03	31.8 ± 2.8
Chitosan-Thyme Essential Oil Nanocapsules (TEO-CSNC)	139 ± 1	0.30 ± 0.01	8.03 ± 1.1

**Table 2:** Constants and regression coefficients for models applied to the release profile in chitosan-thyme nanocapsules.

Cero-Order		First-Order		Higuchi		Korsmery and Peppas		
K_0_	R^2^	K_1_	R^2^	k_H_	R^2^	n	k	R^2^
0.097	0.667	0.089	0.746	0.124	0.968	0.193	0.97	0.991

**Table 3:** Evolution of PS, PDI, ζ and residual content of TEO-CSNC during storage at 4 and 25 °C.

Week	4 °C				25 °C			
	PS (nm)	PDI (-)	*ζ* (mV)	RC (%)	PS (nm)	PDI (-)	*ζ* (mV)	RC (%)
0	139 ± 1 ^a^	0.30 ± 0.01 ^a^	8.0 ± 1.3 ^a^	99.2 ± 0.7 ^a^	139 ± 1 ^a^	0.30 ± 0.01 ^a^	8.0 ± 1.3 ^a^	99.2 ± 0.7 ^a^
1	139 ± 4 ^a^	0.30 ± 0.04 ^a^	10.8 ± 1.6 ^b^	89.4 ± 2.8 ^b^	157 ± 1 ^b^	0.27 ± 0.03 ^a^	20.0 ± 1.0 ^b^	80.1 ± 1.7 ^b^
2	162 ± 3 ^b^	0.32 ± 0.02 ^a^	16.6 ± 0.5 ^c^	79.9 ± 1.1 ^c^	185 ± 2 ^c^	0.32 ± 0.01 ^a^	16.6 ± 0.7 ^c^	76.6 ± 1.4 ^c^
3	180 ± 7 ^c^	0.49 ± 0.01 ^b^	17.0 ± 0.9 ^d^	76.7 ± 1.2 ^c^	284 ± 5 ^d^	0.34 ± 0.03 ^a^	18.1 ± 0.3 ^d^	77.1 ± 0.8 ^c^
4	182 ± 7 ^c^	0.47 ± 0.01 ^b^	6.8 ± 0.3 ^e^	78.5 ± 4.1 ^c^	242 ± 2 ^e^	0.42 ± 0.01 ^b^	17.6 ± 0.8 ^d^	73.6 ± 2.4 ^c^
5	161 ± 3 ^b^	0.50 ± 0.04 ^b^	−11.1 ± 0.3 ^f^	77.3 ± 5.2 ^c^	193 ± 2 ^f^	0.48 ± 0.02 ^c^	16.3 ± 0.5 ^e^	62.0 ± 3.6 ^d^

PS = particle size; PDI = polydispersity index; ζ = zeta potential; RC = residual TEO content. Different letters to the left represent statistically significant differences (*p* > 0.05).

## Data Availability

Not applicable.
